# Supplementary material for: Comparative Transcriptomic Analysis of Insecticide-Resistant Aedes aegypti from Puerto Rico Reveals Insecticide-Specific Patterns of Gene Expression
Source: Genes (Basel). 2023 Aug 15;14(8):1626. doi: 10.3390/genes14081626 (PMC10454789; doi:10.3390/genes14081626)
Supplement: Supplementary file 1 [file genes-14-01626-s001.zip › genes-2521704-Supplementary figures.pdf]

Supplementary Figures

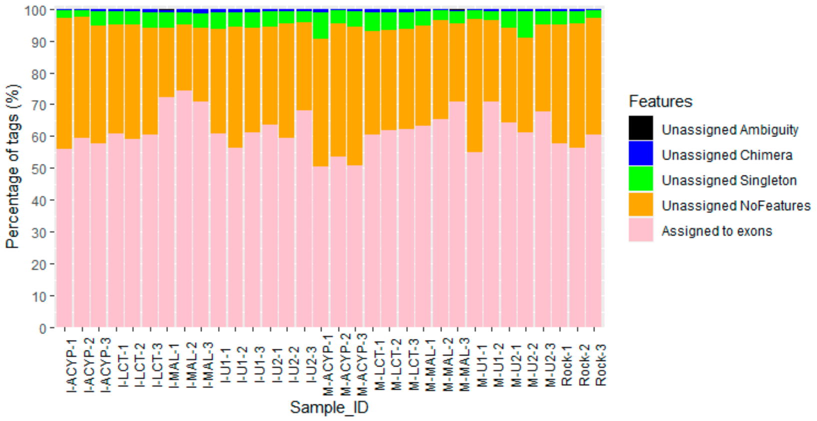

Figure S1: Bar plot of the feature count results. Percentage shows the number of tags successfully mapped to exonic regions (pink) and non exonic region (orange) of the reference genome, as well as those that were not assigned to exons because they were tagged as singleton (green), chimera (blue) and ambiguous (black) alignments.

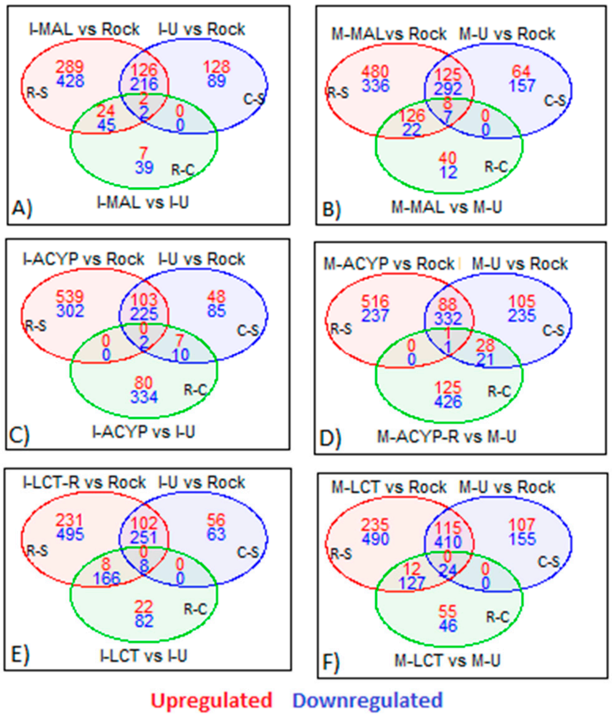

Figure S2: Venn diagrams summarizing the number of differentially expressed genes (DE) and the possible sharing of DE genes in the resistant samples vs susceptible (R-S), control vs susceptible (C-S) and resistant vs control (R-C) for each experiment including Isabela malathion

(A), Manatí malathion (B), Isabela alpha-cypermethrin (C), Manatí alpha-cypermethrin (D), Isabela lambda-cyhalothrin (E) and Manatí lambda-cyhalothrin (F)

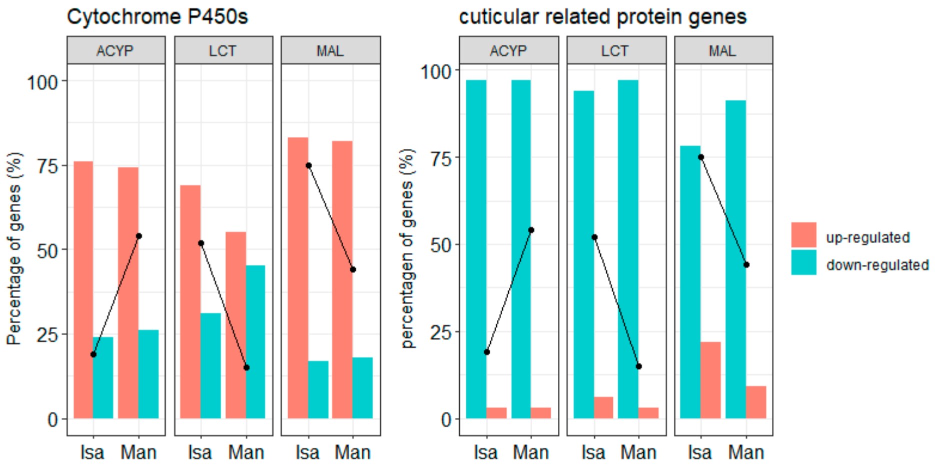

Figure S3: Bar plot showing the percentage of significant up- and downregulated cytochrome P450s (left panel) and the cuticular related protein genes (left panel) in the resistant samples, compared the susceptible strain. Each bar plot is divided into three groups, representing the three insecticides (ACYP, LCT and MAL). No consistent patterns were found between the number of differentially expressed CYP450s, cuticular protein genes, and the survival rate of the mosquitoes to the insecticides.

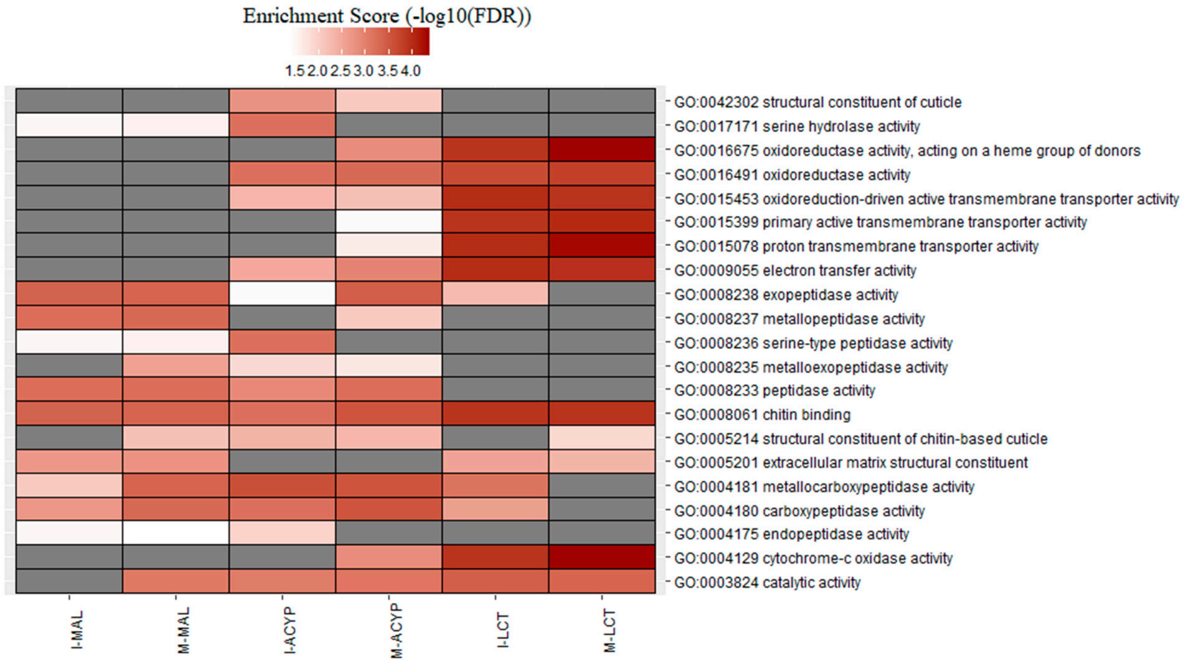

Figure S4: Heatmap of the gene ontology (GO) enrichment analysis of the significantly down-regulated genes in the malathion (I-MAL and M-MAL), alpha-cypermethrin (I-ACYP and -M-CYP) and lambda-cyhalothrin (I-LCT and M-MLCT) resistant samples compared to the susceptible strain of *Ae. aegypti*. The white-red gradient indicates the enrichment score of each GO term, while the gray color indicates that this GO term was not significantly enriched. Only

the core enriched GO-terms (GO terms overlapping across both MAN and ISA) for each insecticide are shown.

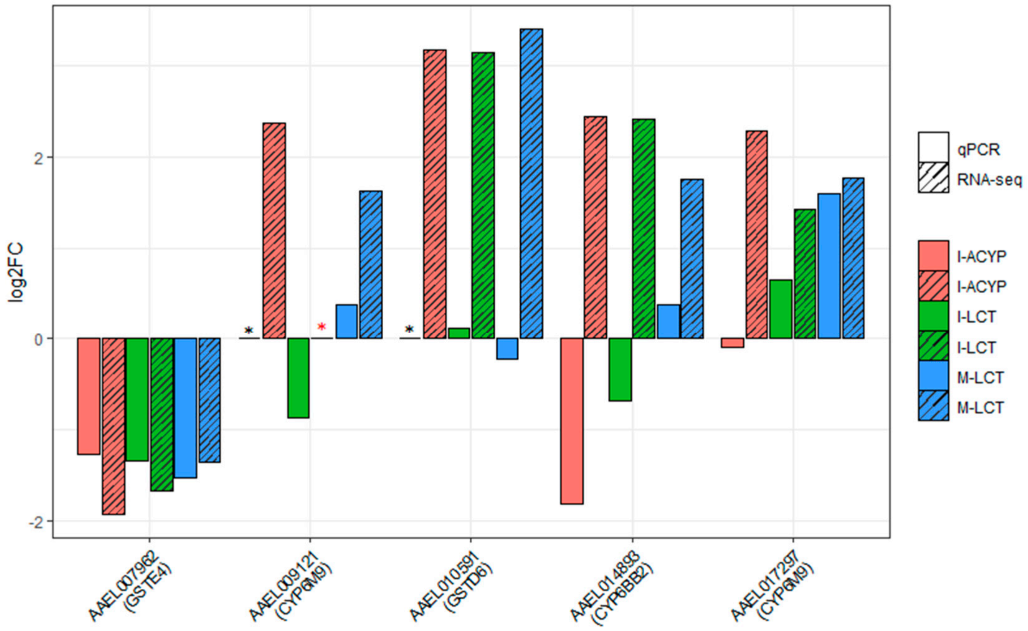

Figure S5: qPCR validation. Comparison of the log2FC expression of 5 detoxification genes measured by qPCR and RNA-seq for the insecticide resistant samples compared to the susceptible. The colors of the bars indicate the type of the pairwise comparison (insecticide resistant vs susceptible) and the patterns of the bars indicate the gene expression measurement approach (qPCR and RNA-seq). The red and the black asterisks (*) indicate genes for which the expression was not detected for the qPCR and the RNA-seq, respectively.
